# Exercise-Induced Elevated BDNF Concentration Seems to Prevent Cognitive Impairment after Acute Exposure to Moderate Normobaric Hypoxia among Young Men

**DOI:** 10.3390/ijerph20043629

**Published:** 2023-02-18

**Authors:** Maciej Chroboczek, Sylwester Kujach, Marcin Łuszczyk, Hideaki Soya, Radosław Laskowski

**Affiliations:** 1 Department of Physiology, Gdansk University of Physical Education and Sport, 80-336 Gdansk, Poland; 2 Sports Neuroscience Division, Advanced Research Initiative for Human High Performance, Faculty of Health and Sport Sciences, University of Tsukuba, Tsukuba 305-8574, Japan; 3 Laboratory of Exercise Biochemistry and Neuroendocrinology, Department of Sports Neuroscience, Advanced Research Initiative for Human High Performance (ARIHHP), Faculty of Health and Sports Sciences, University of Tsukuba, Tsukuba 305-8574, Japan

**Keywords:** physical exercise, cognition, executive functions, altitude

## Abstract

Memory impairment, reduced learning ability, decreased concentration, and psychomotor performance can be all signs of deleterious impact of hypoxia on cognitive functioning. In turn, physical exercise can improve performance and enhance cognitive functions. The purpose of this study was to investigate whether the potential positive effects of exercise performed under normobaric hypoxia can counteract the negative effects of hypoxia on cognitive function, and whether these changes correlate with brain-derived neurotrophic factor (BDNF) concentrations. Seventeen healthy subjects participated in a crossover study where they performed two sessions of single breathing bouts combined with moderate intensity exercise under two conditions: normoxia (NOR EX) and normobaric hypoxia (NH EX). To assess cognitive function, Stroop test was applied. There were no significant differences in any part of the Stroop interference test regardless of the conditions (NOR, NH), despite a statistical decrease in SpO_2_ (*p* < 0.0001) under normobaric hypoxic conditions. In addition, a statistical increase (*p* < 0.0001) in BDNF concentration was observed after both conditions. Acute exercise under normobaric hypoxia did not impair cognitive function despite a significant decrease in SpO_2_. Exercise in such conditions may offset the negative effects of hypoxia alone on cognitive function. This may be related to the significant increase in BDNF concentration and, as a consequence, positively affect the executive functions.

## 1. Introduction

Low- to moderate-intensity exercise in normoxia enhances psychomotor function [1,2,3], while some studies suggest that high-intensity exhausting exercise decreases psychomotor performance [4,5]. Thus, it seems that under normoxia, the exercise intensity can determine its effects. Conditions of hypoxia are more complex. It has been shown that exposure to hypoxia itself usually causes cognitive decline, and this is related to either the time of exposure, the level of hypoxia, or both [6,7].

There are data claiming that hypoxic conditions more likely have a negative impact on central executive tasks (such as executive function) than on non-executive tasks (such as perception, attention, and short-term memory tasks) [8]. However, a meta-regression analysis by McMorris et al. [9] indicated that there is no difference in the effects of hypoxia on cognitive performance between executive and non-executive tasks. Working memory tasks impact the dorsolateral prefrontal cortex, anterior cingulate cortex hippocampus, and maybe the basal ganglia and cerebellum, according to a positron emission tomography study [10]. Therefore, it is believed that certain professions and tasks are more vulnerable to the harmful effects of stress. However, when exercise intensity is moderate, they could also profit from rises in arousal and exercise-enhanced central nervous system arousal, which can be associated with an overall increase in neuroelectric activation [11]. There is also relatively little information regarding the effects of hypoxia on psychomotor performance in subjects who conducted low- and high-intensity exercise. Some evidence suggests that hypoxia impairs cognitive function [6]. Furthermore, the mechanisms of hypoxia’s influence on the central nervous system (CNS) are largely unknown. It is unclear whether the aforementioned exercise outcomes can be influenced by a hypoxia-induced negative impact on the CNS. The brain’s induced release of catecholamines is thought to be mostly responsible for processing-speed increase [12]. Along with increasing levels of central arousal, peripheral catecholamines can also facilitate the production of other stress hormones, raise heart rate, and cause differential vasoconstriction that favors blood flow to skeletal muscle [13]. As the body exerts itself more, each of these highly functional outcomes helps to maximize neurological, metabolic, and muscular performance. When these effects are coupled, they act as both a rapid adaptation to the higher metabolic and functional demands of exercise and a faster, more effective way for the brain to process information centrally [13]. Recent evidence suggests that brain-derived neurotrophic factor (BDNF) plays an important role in this phenomena [14,15]. It has been demonstrated that cognitive impairment occurs in neurodegenerative disorders and is linked to a reduced blood BDNF [16]. Moreover, the magnitude of this decline is dependent on the severity of cognitive impairment [16]. BDNF, on the other hand, is hypothesized to be involved in the improvement of cognitive function as a result of exercise [17]. The rise in BDNF is inversely correlated with exercise intensity and is sensitive to an acute bout of exercise [18]. The inference is that these changes in the serum levels of BDNF are suggestive of increases in BDNF in the brain and, thus, have long-term relevance for brain health and cognitive performance because BDNF is able to pass the blood–brain barrier [19]. In a meta-analysis conducted by Chang et al. [20], executive functions were enhanced during and after acute exercise in normoxia, which is in line with the results of the latest review taken by Basso and Suzuki [21]. Thus, by enhancing synaptic strength, hypoxia-induced BDNF synthesis may benefit memory (i.e., plasticity). BDNF appears to be crucial in the neural pattern separation of comparable information, which has implications for minimizing memory interference, in addition to being associated with improvements in episodic memory performance [22].

A different concept that could explain at least some of the decreased exercise performance in hypoxic conditions focuses on central fatigue [23]. Reduced brain O_2_ delivery has been shown to decrease cognitive ability [6], which supports this hypothesis. Several investigations [24,25] have found that moderate levels of hypoxia impair psychomotor performance, with low oxygen partial pressure (PaO_2_) being the primary source of cognitive impairment regardless of the type of hypoxia [9].

Another concept that could explain changes in executive functions in response to exertion is the cardiac-locomotor synchronization (CLS). Synchronization phenomena occur [3] where two oscillators with distinct periodicities are persuaded to oscillate “at the same step” and become entangled. It was hypothesized that CLS could enhance the blood flow to muscles during contraction to reduce skeletal muscle oxygen deprivation and/or reduce the energy cost of cardiac muscle contraction [26]. Thus, more complicated motor activities can be realized because of this cognitive–motor integration [27]. Physiological signal coupling is frequently studied in the present day in order to further our understanding of motor control, beginning with sensorimotor integration [28]. Research on muscle synergies implies that muscle activity may be adapted to task-specific biomechanical requirements [29]. Due to these factors, analyzing the gait rhythm provides insight into the patient’s cognitive health. In this relation, studies focusing on the dual tasks condition (DT) have made clear that the challenge of giving equal attention to each activity at the same time can significantly increase the risk of falling [30] and decrease the walking speed, cadence, or the rhythm of the gait [31]

The purpose of this study was to verify whether acute moderate exercise in normobaric hypoxia conditions would affect executive functions, peripheral BDNF concentrations, and whether there would be a correlation between them. We hypothesized that executive functions would improve after intervention as a result of an increase in BDNF concentration. We examined how executive function was affected after acute exercise in normoxic, as well as in normobaric hypoxic, conditions (FIO_2_ = 13%).

## 2. Materials and Methods

### 2.1. Sample Size Analysis

The sample size calculation was carried out by G*Power 3. The goal of this study was to find a relationship between BDNF and cognitive functions with a medium effect size (f = 0.2526456) based on partial eta-squared 0.06. Using the analysis of variance (ANOVA) for repeated measures within factors, setting the α-error to 0.05, the power to 80%, and 2 groups, the minimal sample size was estimated at 34. For this trial, a total of 20 participants were enlisted, accounting for a dropout rate of 10% [32].

### 2.2. Participants

This study included 20 healthy, male participants with moderate physical activity and without a professional sports history. Smoking, a history of high altitude expeditions, dyslexia, daltonism, and impaired vision were among the exclusion criteria for study participation. Subjects did not have any medical contraindications. All volunteers participated in a crossover study where they performed two sessions of single breathing bouts combined with moderate intensity exercise under two conditions (normoxia (NOR EX) and normobaric hypoxia (NH EX)) on different days. Due to injury (n = 1), illness (n = 1), and personal reasons (n = 1), 3 people dropped out of the experiment and 17 individuals were subjected to the final analysis. Participants underwent familiarization on all laboratory equipment a week before the intervention and were randomly assigned to one of two groups. The experiment started on the next visit. Participants performed 15 min of exercise (50% VO_2peak_) in one of two conditions: (a) breathing ambient air or (b) breathing hypoxic air (fraction of inspired oxygen (FIO_2_) = 0.135)), which corresponded to a simulated altitude of 3500 m above sea level (a.s.l.), depending on the group assignment. In the next session, followed by a 2 week break, they performed the exercises under the opposite conditions. Before and after both sessions, to measure cognitive control, participants performed color-word Stroop task and after that they were subjected to a blood draw. Their SpO_2_ level was monitored during the sessions. To evaluate serum concentration of BDNF, the ELISA method was applied (details in ‘Blood sampling’ section). Written consent was obtained from participants before executing the intervention. In accordance with the Helsinki Declaration, the research was approved by the Local Ethics Committee and the Bioethical Committee of the Regional Medical Society (KB-9/16). Table 1 displays detailed participants characteristics.

### 2.3. Study Design

**Figure 1:**
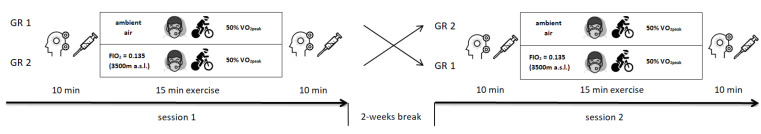
Overview of the experimental protocol.

Participants visited the laboratory a total of three times. A week before the experiment began, participants were requested to attend the laboratory to familiarize themselves with the whole procedure. During intervention sessions participants performed the identical battery of body composition, cognitive tests, and blood collection. Figure 1 provides an overview of the experimental protocol. 

### 2.4. Anthropometric Measurements

A Tanita Body FatMonitor/Scale Analyzer TBF-300 (Japan) was used to estimate body mass and body composition. Body mass index (BMI) (kg·m^−2^) was utilized to assess overall body composition. 

### 2.5. Hypoxic Conditions

The hypoxic gas mixture for the trials was created by the Biomedtech GO2Altitude ERA II Hypoxic/Hyperoxic Air Generator (Australia). The manufacturer’s recommendations were followed in order to reduce the oxygen concentration of the inspiratory gas mixture to replicate height above sea level (a.s.l.), as stated in the GO2Altitude ERA II Hypoxicator System Operational Manual (Biomedtech Australia Pty Ltd., Biomedical Research and Development). The FIO_2_ = 13% oxygen level in the mixture was utilized to construct a hypoxic mix that accurately reflected altitude at 3500 m a.s.l.. Participants were not aware of the gas mixture but were breathing normally. They also wore pulse oximeters and donned masks when doing testing in normoxia, even though the air generator at the time only delivered a sea level breathing mixture [33].

### 2.6. Assessment of Cognitive Performance

The Vienna Test System database’s simplified Stroop interference test was used to assess executive function. Giving “names” for the colors is the first portion of the test. “Reading” color names is discussed in the second part. In the last task, instead of reading the written words, participants must identify the color of the font that was used to compose each word. For instance, the word “blue” should be the reaction to the stimulus of “green” printed in blue font, preventing the inclination to read “green”. A spontaneous, natural reaction must be suppressed in favor of a task that is managed consciously and is bound by the rules in order to accomplish such a work. The time of each test and the interval between the first and last test are two components that are typically included in the test result [34].

### 2.7. Collection of Blood Samples

Blood was drawn from the antecubital vein into vacutainer tubes before and after the intervention in order to evaluate the serum levels of BDNF. The samples were centrifuged for 15 min at 4 °C at 1000× *g*. Since serum analysis is the most widely used method for examining how individual variations in neuropsychiatric, cognitive, and exercise characteristics connect to human growth factors in blood, the serum samples were frozen and stored at 70 °C after separation. Before usage, the sample was diluted 1:5. The intra-assay and inter-assay coefficients of variability (CVs) were reported by the manufacturer to be 3.2–3.0% and 7.2–4.7%, respectively. The manufacturer’s recommendations were followed to perform an enzyme immunoassay using commercially available kits to determine the level of serum BDNF (R & D Systems, Minneapolis, MN, USA, catalogue no. DBNT00; Ray Biotech Inc., Cambridge, UK). A 1 h clotting period was permitted for the proper serum BDNF dosage based on our prior experiences and recommendations in the literature [7,35,36]. 

### 2.8. Statistical Analysis

Microsoft Excel 10.0 for Windows was used for the first archiving of the results. An investigation was then carried out using the statistical analysis capabilities in GraphPad Prism 7. Calculations were made of arithmetic means, standard deviation, and levels of significance for variances between means. After that, the distribution of each variable was analyzed using descriptive statistical techniques, and a parametric paired Student’s *t*-test was run. Afterwards, a two-way analysis of variance (ANOVA) with repeated measurements was used to determine the significance of variances between groups and over time. This was followed by the Bonferroni post hoc test. Additionally, Spearman correlation analysis between BDNF and reaction time in Stroop interference was carried out to highlight the connections between executive functions. For all analyses, significance was set at *p* < 0.05. 

## 3. Results

### 3.1. Blood Saturation during Exercise in Normoxia and Acute Normobaric Hypoxia Conditions

**Figure 2:**
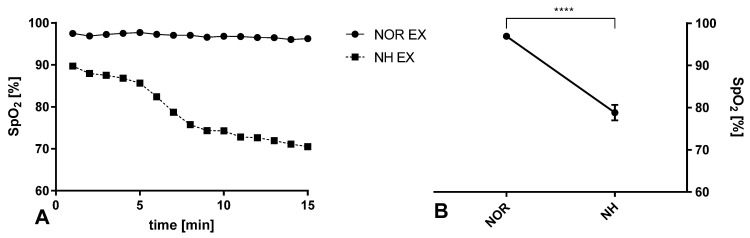
Effect of acute moderate exercise in normoxic and normobaric hypoxic (FIO_2_ = 13%) conditions on peripheral blood saturation. NOR EX—exercise in normoxia; NH EX—exercise in normobaric hypoxia. (**A**) Data expressed as mean of the entire group at particular time points; (**B**) data show differences in means between groups. Error bars indicate ± SEM (standard error of mean). **** *p* < 0.0001.

Blood saturation decreased during acute exposure to normobaric hypoxia (t = 10.51; *p* < 0.0001), and these effects were adequate for the simulated altitude above sea level (Figure 2). 

### 3.2. Stroop Test after Exercise in Normoxia and Acute Normobaric Hypoxia Conditions 

**Figure 3:**
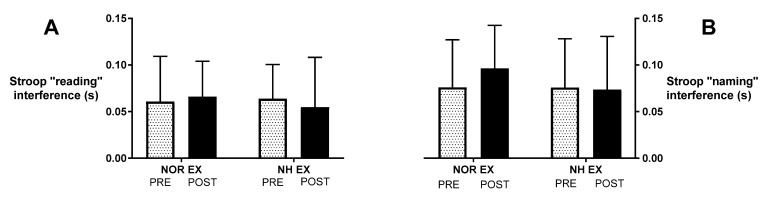
Effect of exercise in normoxia and acute normobaric hypoxia (FIO_2_ = 13%) conditions on post-exposure interference values in reading (**A**) and in naming (**B**). Values are means. Error bars indicate ± SD (standard deviation).

After exercise in both normoxic and at 3500 m a.s.l. simulated altitude conditions, participants underwent cognitive tests. The results are provided in Table 2 and Figure 3. There were no statistically significant changes in either “reading” (interaction F(1, 32) < 1, *p* = 0.49; time F(1, 32) < 1, *p* = 0.85, η^2^ = 0.001) (Figure 3A) or “naming” interference values (interaction F(1, 32) = 1.7, *p* = 0.20; time F(1, 32) = 1.15, *p* = 0.29, η^2^ = 0.012) (Figure 3B), despite a significant drop in saturation under hypoxic conditions. 

### 3.3. Blood Analysis

**Figure 4:**
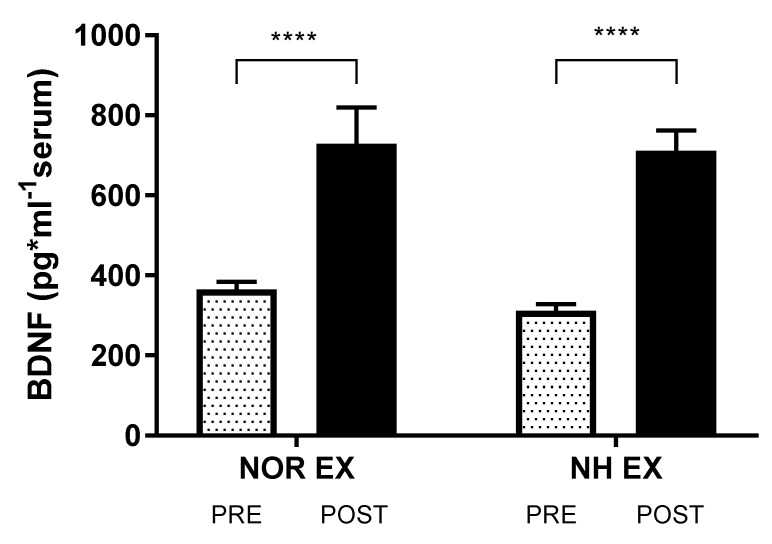
Effect of exercise in normoxia and acute normobaric hypoxia (FIO_2_ = 13%) conditions on post-exposure serum BDNF concentration. Values are means. Error bars indicate ± SD. **** *p* < 0.0001.

Pre–post assessments of the mean levels of BDNF concentration are provided in Table 2 and Figure 4. They showed a significant increase in BDNF concentration following exercise in both conditions (interaction F(1, 32) < 1, *p* = 0.72; time F(1, 32) = 59.45, *p* < 0.0001, η^2^ = 0.553) (Figure 4).

**Figure 5:**
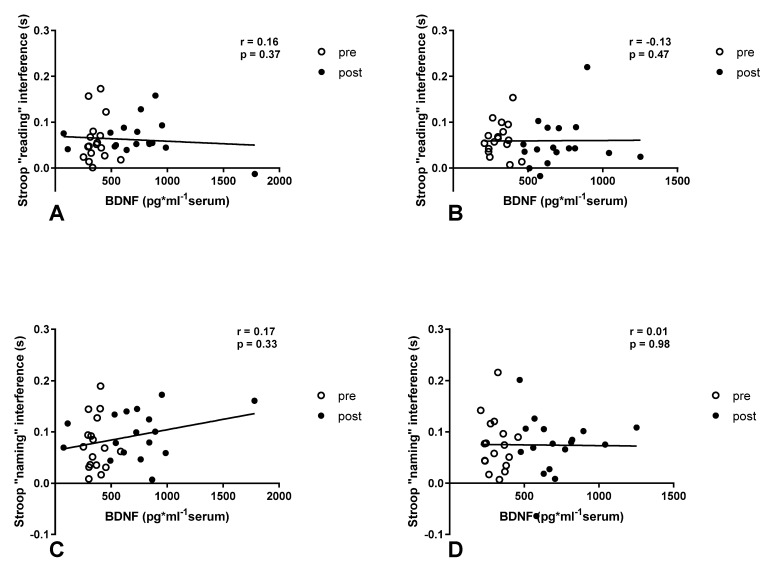
Correlation between Stroop interference and BDNF concentration after acute bout of exercise in normoxia (**A**,**C**) and normobaric hypoxia at FIO_2_ = 13% (**B**,**D**).

The relationship between BDNF concentration and Stroop interference was also investigated. There were no significant correlations between the mentioned variables in either normoxia or simulated acute normobaric hypoxia conditions (Figure 5).

## 4. Discussion

This experiment is part of a larger project in which previous results have shown negative effects of hypoxic breathing sessions on cognitive functions [7]. The main result of this work was a significant increase in BDNF concentration after a single bout of exercise in normobaric hypoxic conditions with a corresponding lack of deterioration in executive functions, even though this impairment was expected based on previous results. The lack of deterioration of these functions, despite adverse environmental factors affecting sensitive brain structures, may be related to increased BDNF as one of the regulators of the response to hypoxia and also as a factor associated with cognitive function.

Recent review has suggested that exposure to hypoxia may impair cognitive functions [9]. Other meta-analysis supports these results by showing that hypoxia had a selective impact on cognition, improving information processing but impairing cognition-based attention, executive function, and memory [37]. Moreover, there are suggestions that the detrimental impact on cognitive performance in hypoxia can be explained, at least partially, by a concurrent drop in BDNF [38]. According to previous studies, SpO_2_ gradually drops as hypoxia becomes more severe [39], and low SpO_2_ levels can lead to brain deoxygenation [40]. Therefore, it is conceivable that hypoxia, as a result of neurological and structural changes to the brain tissue, may be to blame for adverse cognitive-related results [41]. Considering that acute exercise in hypoxic conditions causes progressive brain desaturation [42], cognitive enhancement in such an environment may be diminished as desaturation progresses. In fact, those who showed a higher drop in SpO_2_ during such conditions noted lower cognitive benefits [43]. 

On the other hand, there are studies which reported that the type of exercise, the participants’ level of physical fitness, and the timing of the cognitive task—during or after exercise—all affect the impact of acute exercise on cognitive function [20,44]. For instance, a meta-analysis conducted by Lambourne and Tomporowski [44] reported detrimental impact on cognitive performance during exercise, while beneficial effects were seen after exercise. The timing of testing immediately following the intervention may be the key factor. The reperfusion phenomenon that occurs after oxygen/saturation is reduced may result in increased blood flow through areas in the brain. These areas are supplied with fresh blood that is rich in oxygen and, in addition, with increased BDNF as an underlying factor in exercise/training-induced positive effects on CNS [20,45], which may result in an inhibition of cognitive decline due to hypoxia. Furthermore, in living humans, central BDNF cannot be quantified. The brain is thought to be the primary source of the elevated BDNF in circulation [46]. In our research, both experimental groups had comparable serumconcentrations of BDNF, but cognitive performance was not affected by hypoxia, which has been noted in other studies [47]. The impact of the exercise itself and its intensity seems to also be important. Moderate-intensity exercise in a hypoxic conditions improves cognitive performance, which was prominent in other studies [48,49]. Moderate- intensity exercise may increase cerebral blood flow and compensate for decreased SpO_2_, when exposed to hypoxia [50]_._ This finding could be related to the possible combined effects of moderate hypoxia and exercise on cognition. Exercise and moderate levels of hypoxia may improve synaptic plasticity by increasing the expression of BDNF, as thoroughly explained elsewhere [51,52]. BDNF can be upregulated by hypoxia, as well as by exercise, which can promote cerebral neuronal activation and neurogenesis and, as a result, enhance cognition [53]. The evidence is strong that specific dual-task training programs can significantly improve patients’ executive functions, which are positively impacted by aerobic exercises [54]. It makes sense that people would be able to maintain the effectiveness of resource allocation on cognitive–physical dual task performance when coordination and aerobic training is combined with multi-task exercise [55]. It is impossible to emphasize how crucial decision-making can be, and it goes without saying that (i.e., in sports) decisions must be made both during and right after physical activity of varying intensities. According to information processing specialists, making decisions (i.e., in team games) requires the player to accurately perceive the environment, store that perception in short-term memory, and contrast the current environment with previously learned lessons that are stored in long-term memory. It is also known from the meta-analysis that high aerobic fitness is not necessary for transient enhancements of the executive control system, and both low- and high-fit people appear to gain equivalent benefits from exercise [56]. Therefore, in order to prepare strategically for a circumstance requiring strong executive control, we can perform such aerobic exercise sessions to obtain better results.

A few limitations of this study must be mentioned. In the future, study groups may be larger in order to examine sex or age differences in terms of executive functions after exercise. Saturation could also be measured post-exercise (if this would not impede the participants cognitive test performance). In future studies, it would also be worth including measurements of the brain tissue oxygenation to examine the various mechanisms causing these alterations. Likewise, other types of exercise with equivalent intensity or chronic training intervention should be taken into account when planning future studies.

It is suggested that the combination of exercise duration and intensity, the severity of hypoxia, and the exposure time is what primarily determines the effects of acute exercise and hypoxia on cognitive performance.

## 5. Conclusions

Although the effects of acute hypoxia on cognitive function are still up for debate, hypoxia can affect cognition. On the other hand, exercise can lead to improvements in cognitive abilities. The effects of a single bout of exercise with exposure to simulated moderate normobaric hypoxia conditions were investigated and no differences were observed. This exposure was accompanied by a rise in BDNF and a decreased SpO_2_ level. Higher BDNF levels can improve human cognition and cause beneficial changes; in these circumstances, no negative impacts on cognition were seen. Further research focusing on training in such conditions and its effect on cognition need to be done. These results suggest that acute moderate exercise has beneficial effects that may protect cognitive processes from the adverse consequences of hypoxia, especially in terms of cognitive functions, which are extremely important in both young people and the elderly because they can improve learning and memory. There is substantial evidence that certain dual-task training programs can help improve executive functions, which could be benefited by aerobic exercise.

## Figures and Tables

**Table 1:** Participants characteristics.

N = 17	X	SD
Age [years]	20.6	0.7
Weight [kg]	75.6	8.3
FAT [%]	17.9	2.9
FAT [kg]	13.7	3.1
FFM [kg]	62.0	6.3
BMI [kg∙m^−2^]	23.3	2.0
VO_2max_ [mL∙kg^−1^∙min^−1^]	42.1	6.5

X—mean average; SD—standard deviation; FAT—adipose tissue; FFM—free fat mass; BMI—body mass index.

**Table 2:** Effect of intervention on BDNF concentration and Stroop interference results.

	NOR EX (n = 17) Mean ± SD	NH EX (n = 17) Mean ± SD	Diff	95% CI		*p*
				Lower	Upper	
**BDNF (pg·mL^−1^ serum)**						
Before	364.19 ± 80.72	311.08 ± 70.4	53.11	−121.1	227.3	0.9731
After	728.35 ± 375.54	711.38 ± 207.96	16.97	−157.2	191.1	>0.9999
Change	364.2 ± 359.1	400.3 ± 195.4				
*p*	<0.0001	<0.0001				
**Stroop “reading” interference (s)**						
Before	0.06071 ± 0.04863	0.06385 ± 0.03673	−0.003147	−0.03842	0.03213	>0.9999
After	0.066 ± 0.03805	0.05465 ± 0.05356	0.01135	−0.2392	0.04663	0.9256
Change	0.005294 ± 0.04917	−0.009206 ± 0.0689				
*p*	>0.9999	>0.9999				
**Stroop “naming” interference (s)**						
Before	0.07588 ± 0.0512	0.07576 ± 0.05245	0.0001176	−0.0407	0.04094	>0.9999
After	0.09632 ± 0.04619	0.07371 ± 0.05698	0.02262	−0.0182	0.06344	0.4161
Change	0.02044 ± 0.04423	−0.002059 ± 0.0553				
*p*	0.2041	>0.9999				

## Data Availability

The data presented in this study are available on reasonable request from the corresponding author.
